# Bestrophin-3 Expression in a Subpopulation of Astrocytes in the Neonatal Brain After Hypoxic-Ischemic Injury

**DOI:** 10.3389/fphys.2019.00023

**Published:** 2019-01-29

**Authors:** Veronika Golubinskaya, Regina Vontell, Veena Supramaniam, Josephine Wyatt-Ashmead, Helena Gustafsson, Carina Mallard, Holger Nilsson

**Affiliations:** ^1^Department of Physiology, Institute of Neuroscience and Physiology, The Sahlgrenska Academy, University of Gothenburg, Gothenburg, Sweden; ^2^Division of Imaging Sciences & Biomedical Engineering, Centre for the Developing Brain, King’s College London, King’s Health Partners, St Thomas’ Hospital, London, United Kingdom; ^3^Wigglesworth Perinatal-Padiatric Pathology Service, Imperial College Healthcare NHS Trust, London, United Kingdom

**Keywords:** bestrophin-3 (Best3), alternative splicing, astroglia, endoplasmic reticulum (ER) stress, hypoxia-ischemia

## Abstract

Bestrophin-3, a potential candidate for a calcium-activated chloride channel, recently was suggested to have cell-protective functions. We studied the expression and alternative splicing of bestrophin-3 in neonatal mouse brain and after hypoxic-ischemic (HI) injury and in human neonatal brain samples. HI brain injury was induced in 9-day old mice by unilateral permanent common carotid artery occlusion in combination with exposure to 10% oxygen for 50 min. Endoplasmic reticulum stress was induced by thapsigargin treatment in primary culture of mouse brain astrocytes. We also investigated expression of bestrophin-3 protein in a sample of human neonatal brain tissue. Bestrophin-3 protein expression was detected with immunohistochemical methods and western blot; mRNA expression and splicing were analyzed by RT-PCR. HI induced a brain tissue infarct and a pronounced increase in the endoplasmic reticulum-associated marker CHOP. Three days after HI a population of astrocytes co-expressed bestrophin-3 and nestin in a penumbra-like area of the injured hemisphere. However, total levels of Bestrophin-3 protein in mouse cortex were reduced after injury. Mouse astrocytes in primary culture also expressed bestrophin-3 protein, the amount of which was reduced by endoplasmic reticulum stress. Bestrophin-3 protein was detected in astrocytes in the hippocampal region of the human neonatal brain which had patchy white matter gliosis and neuronal loss in the Sommer’s sector of the Ammon’s horn (CA1). Analysis of bestrophin-3 mRNA in mouse brain with and without injury showed the presence of two truncated spliced variants, but no full-length mRNA. Total amount of bestrophin-3 mRNA increased after HI, but showed only minor injury-related change. However, the splice variants of bestrophin-3 mRNA were differentially regulated after HI depending on the presence of tissue injury. Our results show that bestrophin-3 is expressed in neonatal mouse brain after injury and in the human neonatal brain with pathology. In mouse brain bestrophin-3 protein is upregulated in a specific astrocyte population after injury and is co-expressed with nestin. Splice variants of bestrophin-3 mRNA respond differently to HI, which might indicate their different roles in tissue injury.

## Introduction

Bestrophins are anion channels that generally serve as chloride channels involved in regulation of cell excitability or cell volume regulation (Duran et al., 2010). Mainly three isoforms have been identified, and on the mRNA level all have been shown to be expressed in the brain (Krämer et al., 2004). Best1 is the most studied, and can function not only as a chloride channel, but also as a release mechanism for glutamate (Oh et al., 2012; Han et al., 2013) and GABA (Park et al., 2009; Lee et al., 2010) in brain astrocytes. It seems to be of special importance in reactive astrocytes, where redistribution of Best1 parallels enhanced GABA production (Oh and Lee, 2017). Data on Best2 and Best3 in brain are scarce.

Best3 mRNA is subject to alternative splicing in mouse and human, resulting in a number of splice variants of unclear function. Some splice variants of Best3 have the channel pore region spliced out, likely causing them to lose ability to act as ion channels (Srivastava et al., 2008). A specific splice variant has been suggested to control intracellular calcium release in myoblasts, possibly preventing calcium overload (Wu et al., 2016). Best3 has recently also been implicated in various cell-protective mechanisms outside the brain. This mainly stems from three studies: (a) Best3 has anti-apoptotic function in basilar artery smooth muscle cells stressed by hydrogen peroxide (Jiang et al., 2013), (b) Best3 ameliorates TNFα-induced inflammation in endothelial cells (Song et al., 2014), and (c) Best3 decides cell fate in an endoplasmic reticulum (ER) stress model in renal proximal tubule cells (Lee et al., 2012).

One of the consequences of cerebral ischemia is ER stress and the unfolded protein response (UPR) in brain cells. These processes have a complex role in cell injury, promoting either survival or death of the cells in the brain, depending on injury conditions (DeGracia and Montie, 2004). ER stress has been demonstrated also in astrocytes, resulting in increased production of cytokines and less neurotropic factors (Singh et al., 2018), potentially enhancing tissue injury. At the same time ER stress can have a protective preconditioning effect in cell injury (Lehotský et al., 2009; Leonard et al., 2014). Both ER stress (Chavez-Valdez et al., 2016) and apoptosis (Zhu et al., 2005) are important mechanisms following injury in the developing brain.

The role of Best3 in brain injury is unknown. Since Best3 has been implicated in cell protection after ER stress, we hypothesized that it could serve as a marker for cells involved in the response to brain injury. We used a well-established model of neonatal hypoxia-ischemia in mice. Unilateral brain injury was induced by a combination of hypoxia and ischemia (HI) (Rice et al., 1981; Sheldon et al., 1998; Hedtjärn et al., 2002). The resultant brain injury is characterized by increased gliosis and apoptosis-dependent brain injury (Blomgren et al., 2001).

Our work shows for the first time the expression of Best3 in neonatal brain in mice and humans, and describes changes in Best3 expression and alternative splicing following brain injury.

## Materials and Methods

### Animals

C57Bl6J mice were obtained from the Jackson Laboratory, United States, and bred in-house at the laboratory of Experimental Biomedicine (EBM) at Sahlgrenska Academy, University of Gothenburg, Sweden.

In total 76 mouse pups of either sex were used in the experiments. Animals were randomly allocated into groups, and different animals were used for mRNA and protein expression analysis. For the mRNA expression analysis, a total of 41 pups (21 male, 20 females) were used, among those 26 pups in the HI group (12 males, 14 females) and 15 pups in the sham-operated group (9 males, 6 females). These were divided into separate groups for the various time points. The 6 h time point: 5 pups in the HI group (3 males, 2 females) and 3 pups in the sham group (2 males, 1 female); 12 h time point: HI 6 pups (3 males, 3 females), sham 4 pups (2 males, 2 females); 24 h time point: HI 6 pups (3 males, 3 females), sham 3 pups (2 males, 1 female); 72 h time point: HI 5 pups (2 males, 3 females), sham 3 pups (1 male, 2 females); 7 days time point: HI 4 pups (1 male, 3 females), sham 2 pups (2 males). For protein expression analysis by western blot a total of 18 pups were used, HI 14 pups (8 males, 6 females), sham 4 pups (2 males and 2 females). In experiments with immunostaining 9 pups of either sex were used. In experiments with astrocyte primary culture 8 pups of either sex were used.

Animals were euthanised either by an overdose of barbiturates or by direct decapitation (in experiments with primary cell culture of brain cells). All animal experiments and methods for animal euthanasia were approved by the Animal Ethics Committee in Gothenburg.

### Model of Neonatal Hypoxia-Ischemia (HI) Injury

Nine-day old mice of either sex were exposed to HI treatment according to the Rice-Vanucci model (Rice et al., 1981; Sheldon et al., 1998). Under isoflurane anesthesia (1.5% in a 1:1 mixture of N_2_ and O_2_) the left common carotid artery was permanently ligated with proline 6.0 suture, then the incision was closed and treated with local anesthetic. The pups were returned to their mothers for 1 h recovery. After that pups were placed for 50 min in a 36° C -heated incubator containing a humidified gas mixture of O_2_ (10%) in N_2_. Subsequently pups stayed with their mothers until removed for tissue sampling. No mortality was observed after HI under the conditions used. Control sham-operated animals underwent the same surgical procedure except for the ligation of the carotid artery, and later they were kept under warm conditions in normal air at the same time as HI treatment was performed. Animals were euthanised at the time points of 6, 12, 24 and 72 h after HI.

In this model, the left hemisphere, ipsilateral to the artery ligation, receives a reduced blood flow, which in combination with hypoxia results in unilateral injury to this hemisphere. The contralateral (right) hemisphere does not experience a fall in cerebral blood flow (Ek et al., 2015), and no gross morphological injury is observed in this hemisphere (Vannucci and Hagberg, 2004). Left and right hemispheres from sham-operated mice were used as non-treated controls.

### Primary Culture of Mouse Brain Astrocytes

Experiments were based on previously described procedures for primary culture of murine brain cells (Lund et al., 2006; de Vellis et al., 2010). 1–2 days old mouse pups were decapitated, the brain was quickly removed and washed in cold Hanks’ balanced salt solution (HBSS, Sigma-Aldrich) containing 1% antibiotics (penicillin-streptomycin, Sigma-Aldrich). Then the cerebellum was removed, and the rest of the brain tissue was mechanically homogenized by pipetting in warm Dulbecco’s modified Eagle’s medium (DMEM, Sigma-Aldrich, D5796) containing 1% antibiotics and 20% fetal bovine serum (FBS, Gibco, Life Technologies). Further culture medium always contained 1% of antibiotics. A homogenate of 1–2 brains was filtered through a 70 μm-diameter filter and diluted up to the final volume of 24 ml in DMEM with 20% FBS, and finally transferred to 75 cm^2^ flask (Sarstedt AB, Helsingborg, Sweden). Cells were left growing in 5% CO_2_/95% air at 37°C for 7 days, then medium was changed to DMEM with 10% FBS, and cells were allowed to grow for an additional week. After that flasks were shaken for 24 h at 250 rpm in 5% CO_2_/95% air at 37°C. The medium was removed, cells were washed 2–3 times with warm HBSS and incubated in 0.05% Trypsin+EDTA (in HBSS) for 20-30 min in 5% CO_2_/95% air at 37°C with gentle shaking, until the cells fully detached from the flask bottom. Then trypsin activity was blocked by adding an equivalent volume of DMEM with 10% FBS. After that cell suspension was transferred to 50 ml tubes and centrifuged at 250 g for 5 min. The supernatant was discarded, the cell pellet was resuspended in DMEM with 10% FBS, and the number of cells per 1 ml was measured (Scepter TM Automated Cell Counter, Merck Millipore). Cells were plated either 50,000 cells per well in 12-well plate (Corning Inc., United States) for further studying the mRNA or protein expression, or 10,000 cells per well in 8-well glass slide chamber (BD Falcon TM culture slides, BD Biosciences, United States) for further immunostaining. Cells were left to attach and grow for 3 days, and then the treatments were performed. The resulting cell culture contained about 95% of GFAP-positive astrocytes, among the remaining cells microglia could be detected (de Vellis et al., 2010).

The ER stress was induced in the cells by treatment with thapsigargin (TG, 200 nM, Sigma-Aldrich) in serum-free DMEM; control cells received the same medium. In this concentration TG induces ER stress in cultured astrocytes and increases ER-stress markers (Johnson et al., 2014). Forty-eight hours after the treatment cells were harvested for mRNA and western blot analysis.

### Preparation of the Samples for Immunostaining in Mouse Brain Tissue

For brain tissue samples the animals were euthanised by an overdose of barbiturates i.p. and immediately transcardially perfused first with saline, then with PFA (Sigma-Aldrich) 4% in PBS. Brains were then removed and kept in 4% PFA for 1 day at 4°C. Then tissues were dehydrated in graded ethanol and xylene substitute Tissue-Clear^®^ (Tissue-Tek^®^ Sacura) and embedded in paraffin. Coronal sections (5 μm) of the brain were prepared and mounted on glass slides.

For immunochemistry in primary astrocyte culture the cell medium was replaced with 4% PFA, and the cells were fixed for 15 min at room temperature and stored in PBS at 4°C for 1–2 weeks until the staining was performed.

### Immunostaining

Immunostaining in mouse brain sections was performed as described in Golubinskaya et al. (2015). In short, PFA-fixed tissue and cell preparations were deparaffinised and received standard antigen retrieval treatment: the preparations on the glass slides were placed in 50 mM citrate buffer (pH 6.0) and heated to 98–99°C for 30 min. Then the preparations were permeabilized and incubated with primary antibody for 24–36 h at 4°C. One of the specimens in each experiment was processed the same way, but without primary antibodies in the incubation buffer, to control for possible non-specific binding of the secondary antibody.

The primary anti-mouse antibodies used were obtained from commercial sources: Best3 (Rabbit polyclonal, IgG, Bst-301AP, lot# A532.Fb.AP, FabGennix International Inc., United States), Nestin (Goat polyclonal, IgG, G-20: sc-21248, Santa Cruz Biotechnology Inc., United States), GFAP (Mouse monoclonal IgG, G3893, Sigma-Aldrich), Iba-1 (Goat polyclonal IgG, orb19198, Biorbyt), NeuN (Mouse monoclonal IgG, MAB377, Millipore). The anti-Best3 antibody does not distinguish between Best3 splice isoforms studied in this paper, as the antigen is localized at the very end of the C-terminal region. An antibody directed to the same region in human Best3 (BEST-312AP, FabGennix), which differs in amino acid sequence, was used as a non-specific IgG control. We have previously shown the specificity of staining with BEST-301AP as it was inhibited by blocking peptide (Golubinskaya et al., 2015).

Secondary antibodies (488 or 594 DyLight-labeled secondary antibody made in donkey, Jackson Immunoresearch, United Kingdom) were incubated with specimens for 60 min at room temperature. Nuclei were detected by DAPI (Invitrogen) in dilution 1:75000. ProLongTM Gold antifade reagent (Invitrogen, Molecular Probes) was used for mounting the slides.

Conventional images were obtained by Olympus BX60 conventional fluorescence microscope (camera Olympus DP50) and Zeiss Imager.Z2 conventional fluorescence microscope (camera AxioCam MRM, Zeiss). Confocal images were obtained with a Zeiss LSM 800 microscope.

### mRNA Expression Analysis

The animals were transcardially perfused with saline. Brains were quickly removed and dissected: cerebellum and the frontal part with olfactory bulbs were removed, then on the coronal projection of the brain the sector containing the cortex and hippocampus injury was dissected out. Tissue was immediately frozen in dry ice and stored for further investigation. Primary astrocyte cells were briefly washed three times with cold PBS and then lysed directly in the wells by RLT buffer (Qiagen GmbH, Germany) containing 1% of beta-mercaptoethanol (Sigma-Aldrich) and then frozen for storage.

For total RNA extraction brain samples were briefly homogenized mechanically on ice in RNAase-free PBS, then a third of the sample was transferred to QIAzol Lysis Reagent (Qiagen GmbH, Germany) and homogenized additionally. Cultured astrocytes were homogenized in beta-mercaptoethanol-containing RLT buffer by intense vortexing. The samples were further processed according to RNeasy Lipid tissue Mini Kit (brain samples) or RNeasy Mini Kit protocol (cultured cells), both with DNAse digestion (QIAGEN GmbH, Germany). Concentration of the extracted total RNA was detected spectrophotometrically as the absorbance at 260 and 280 nm (NanoDrop 1000 spectrophotometer, Wilmington, Del., United States). For cDNA synthesis QuantiTect Rev.Transcription Kit (QIAGEN GmbH, Germany) was used, total amount of RNA used in RT reaction for each sample was 1 μg.

Primers and analysis of PCR products (Figure 2A) were made as described previously (Golubinskaya et al., 2015) in accordance with the predicted mRNA sequence for mouse bestrophin-3 (NM_001007583.1). Also PCR and qPCR analysis of alternative splicing of Best3 mRNA was performed as described by Golubinskaya et al. (2015). For qPCR analysis of Best3, expression primer pairs C (for detecting total mRNA of Best3), G (for variant with exon 6 is present, “+6” variant) and I (for variant missing exon 6, “-6” variant) were used. For quantification of nestin (Mm_Nes_2_SG), CHOP (Mm_Ddit3_2_SG) and a housekeeping gene GAPDH (Mm_Gapdh_3_SG) the commercial primers (QuantiTect Primer Assays from QIAGEN GmbH, Germany) were used.

For each sample the results were calculated as the difference in crossing-point (*C*p) numbers between the housekeeping gene and the gene of interest (delta *C*p). Then from each individual delta *C*p the delta *C*p value of the corresponding control samples was subtracted as shown below (delta delta *C*p). The resulting values are equal to log_2_ of the concentration ratio of the gene of interest between treated and control groups and reflect the change in treated samples in relation to the corresponding control samples.

(1) Effect of HI:
  Log_2_(fold change) = delta *C*p (left hemisphere of HI-treated or sham-operated mouse) - average delta *C*p in the left hemisphere of time point-matched sham-operated mice;
(2) Effect of HI in the uninjured hemisphere:
  Log_2_(fold change) = delta *C*p (right hemisphere of HI-treated mouse or right hemisphere of sham-operated mouse) - average delta *C*p in the right hemisphere of time point-matched sham-operated mice;
(3) Effect of TG in the cell culture:
  Log_2_(fold change) = delta *C*p (sample of treated or control cells) - average delta *C*p in all control samples.

### Protein Expression Analysis in Mouse Brain After HI by Western Blot

Brain tissue was obtained and dissected in the same way as in experiments with mRNA expression analysis. Then the tissue samples were either frozen for further analysis of total protein or were directly homogenized on ice for further subcellular fractionation of proteins. Samples from right (control) and left (injured) hemispheres were processed separately for each animal.

For subcellular fractionation of the proteins the simplified variant of protocol described by Cox and Emili (2006) was used. Briefly, tissue was homogenized in 250-STMDPS buffer (250 mM sucrose, 50 mM TrisBase, 5 mM MgCl_2_, DTT 1 mM, spermine 50 μg/μl, spermidine 50 μg/μl, PMSF 0,1 mM, pH 7.4) in glass Dounce homogenizer tube. The aliquot of homogenate was saved frozen for later total protein analysis. The rest of homogenate was centrifuged at 800 × *g*, 15 min at 4°C, twice after which the supernatant representing the cytoplasmic fraction was saved frozen.

For extraction of total protein from brains after HI, frozen brain tissue and the total protein aliquot from subcellular fractionation were homogenized in the lysis buffer containing Tris-HCl 50 mM, NaCl 150 mM, Triton X-100 1%, Igepal 0,25%, phosphatase inhibitor and protease inhibitor cocktails (Roche, Germany), pH 7.5.

For extraction of total protein from primary astrocytes the cells were washed 2–3 times in PBS, then collected in PBS containing Triton X-100 0.5%, Igepal 0,12%, PMSF 0.1% and protease inhibitor cocktail 0.5% and then stored frozen.

Then all the samples (total protein from HI brains and from primary astrocytes, cytoplasmic and total protein fractions) were additionally mechanically homogenized by vortexing and by passing through 27–29G syringe needle. After that protein concentration in the samples was measured by BCA method. Then samples were prepared in Laemmli sample buffer 4× (Bio-Rad Laboratories, United States) with 10% DTT, heated for 5 min at 95°C and run through the 4–20% Criterion TGS Stain-free gel in amounts of 10 μg/well for total protein and cytoplasmic fractions and 1 μg/well for total protein samples from primary astrocytes. Protein was transferred to 0.2 μm PVDF membrane (Bio-Rad) by Trans-Blot Turbo Transfer System (Bio-Rad). Then membrane was washed in TBS-T buffer, blocked by 5% milk solution and stained with primary antibody against Best3 (5000 dilution in PBS with 3% BSA and 0.1% sodium azide). Bst-301AP (FabGennix International Inc., United States) was used for detection of Best3 in primary astrocyte samples, and ARP50108_P050 (Aviva Systems Biology, United States) was used for analysis of Best3 in HI brain tissue samples. We have previously shown specificity of Western blot detection with Bst-301AP by inhibition with blocking peptide (Matchkov et al., 2008); Supplementary Image S2 here shows the same for ARP50108_P050. For visualization of Best3 peroxidase-labeled secondary antibody (Vector Laboratories, United States) and SuperSignalR West Dura Extended Duration Substrate (Thermo Scientific, United States) were used. The images were captured by ChemiDoc^TM^ Imaging System, Bio-Rad. Precision Plus Unstained and Precision Plus All blue Protein Ladders from Bio-Rad were used to estimate the molecular weight of visualized proteins. Bands were identified and their area-intensity product normalized to total protein using ImageLab software (Bio-Rad).

### Statistical Analysis

In the experiments *n* equals number of animals (brain tissue samples) or wells (cell culture studies). Data of qPCR experiments (delta *C*p values) was analyzed by three-way mixed ANOVA (analyzing brain hemispheres (within subjects), treatment and time (between subjects) using GLM for repeated measurements using IBM SPS Statistics 25; simple effects were analyzed from estimated marginal means with Bonferroni adjustment. Data from western blot were analyzed by two-way mixed ANOVA. Analyses of two groups were by means of Student’s *t*-test. Data is presented on the graphs as individual values with group means ± SEM. A *P*-value of less than 0.05 was regarded as significant. In Figure 3 values for treated animals are plotted as differences from the corresponding means of sham animals; the detailed statistical results for Figure 3 are given in the Supplementary Table S1.

### Immunostaining in Human Neonatal Post-mortem Brain Tissue With Pathology

Informed parental consent was acquired in accordance with the National Health Services United Kingdom guidelines and research study ethics was obtained from the National Research Ethics Services (West London), United Kingdom (ethics number 07/H0707/139; Postmortem Magnetic Imaging Study of the Developing Brain). The postmortem case assessed in this study was a term stillbirth/intrauterine death (at 41.7 weeks gestational age; male) associated with amniotic fluid infection. The pregnancy was otherwise uncomplicated and there were no major congenital anomalies, chromosomal defects or overt culture-positive sepsis evident in the infant. The clinical pathologist noted that there was slight congestion and oedema seen throughout the brain and there was evidence of patchy white matter gliosis. However, except for the hippocampal region which had overt neuronal loss in the Sommer’s sector of the Ammon’s Horn, the rest of the brain was unremarkable (i.e., no hypoxic-ischemic neurons or apoptotic neurons were seen). Samples containing the Ammon’s Horn, thalamus and lentiform nucleus were used in this study. Postmortem tissue preparation has previously been described (Vontell et al., 2015) and the standard immunohistochemistry procedure for neonatal brain tissue has been described elsewhere (Supramaniam et al., 2013; Vontell et al., 2013). The tissue slides were incubated with the human-specific antiBest3 primary antibody (1:200 rabbit polyclonal, ab101828 from Abcam, Cambridge, United Kingdom) overnight, followed by incubation with secondary biotinylated antibody (1:1000 goat-anti-rabbit, BA-1000, Vector Laboratories, Burlingame, CA, United States) for 1 h, and then processed as described in Vontell et al. (2015).

## Results

### Expression of Best3 Is Seen in the Ventricles of Non-injured Mouse Brain

In non-injured mouse brains (in sham-operated animals and in the right, contralateral hemisphere of the brain of HI mice) immunohistochemical analysis revealed presence of Best3 protein in ependymal cells and occasionally in radial-glia on the surface of the brain ventricles (Supplementary Image S1), but no obvious staining in other brain cells was detected. A similar pattern was seen in HI-injured mouse brain (left, ipsilateral hemisphere). We also have a preliminary observation of similar staining for Best3 in brain ependymal cells in the brains of adult naive mice.

### Immunofluorescent Analysis Shows Best3 Protein Expression in Mouse Brain Astrocytes After Injury

**FIGURE 1:**
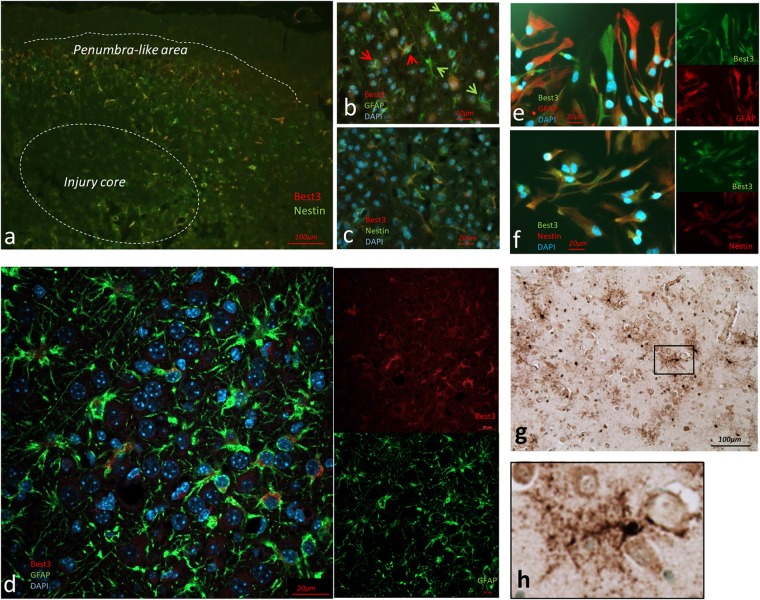
Immunohistochemical evidence for Best3 expression in newborn mouse and human brain after injury. Best3 is expressed in mouse brain cortex after HI **(a–d)**, in mouse primary astrocyte culture **(e,f)** and in brain of newborn child with birth pathology **(g,h)**. Best3 was expressed in the injured mouse brain 3 days after HI **(a)** in a subpopulation of GFAP-positive cells **(b,d)**. Some cells expressed both Best3 and GFAP (**b**, red arrows), while other GFAP-positive cells did not express Best3 (**b**, green arrows). Best3-positive cells also expressed the intermediate filament nestin and were localized mostly in cortex and hippocampus in a penumbra-like area of the injury **(a,c)**. Confocal image of the penumbra-like area **(d)** shows Best3 intracellularly in the perinuclear area. Similar to the brain tissue astrocytes, some, but not all GFAP-positive astrocytes in a primary culture were expressing Best3 **(e)**. These cells also expressed the intermediate filament nestin **(f)**. Best3 is stained red, nestin and GFAP green **(a–d)**, Best3 is stained green, nestin and GFAP red **(e,f)**, nuclear staining with DAPI blue, magnification 10× **(a)**, 40× **(b,c)**, 60× **(d)**. Best3 was also expressed in astrocyte-like cells in cortical areas of the brain of a term infant with white matter gliosis (**g**, enlarged Best3-positive structure in **h**). Peroxidase-based immunostaining captured at magnification 20×.

After HI Best3-positive cells were detected in the injured hemisphere, primarily in the cortex and hippocampus in the penumbra-like area of injury (Figure 1a) and this expression pattern was similar in male and female pups. Best3-positive cells were first seen at 24 h after injury, but the staining was most pronounced 72 h after the HI event. Seven days after injury almost no injury-associated Best3-positive cells were evident. Best3 immunoreactivity was not detected in the contralateral non-injured hemisphere in cortical and hippocampal regions.

Best3-positive cells also expressed the intermediate filament nestin (Figure 1a,c). Best3 expression in astrocytes was supported by the observation that Best3 staining did not overlap with neuronal markers NeuN or calretinin, nor with Iba-1, a marker for microglia. With regard to the astrocyte marker GFAP, it was clear that Best3 was expressed in some but not in all GFAP-positive cells (Figure 1b,d). This suggests that after injury Best3 is expressed in a subpopulation of astrocytes that express nestin. The possibility that some of these cells could be of other type (neural progenitors etc.) cannot be neglected, but the combination of astrocyte markers and cell size and morphology suggests that the majority of Best3-positive cells were astrocytes.

### Immunofluorescent Analysis Shows Best3 Protein Expression in Mouse Astrocytes in Primary Culture

Best3 expression in brain astrocytes was further confirmed by Best3-positive staining in a primary culture of mouse astrocytes (Figure 1e,f). As in the brain tissue, Best3 in cultured astrocytes was co-expressed with nestin (Figure 1h), while overlap with GFAP was not complete (Figure 1e).

### Expression of Best3 Is Seen in the Human Brain

Immunohistochemical analysis showed expression of Best3 protein in astrocytes (Figure 1g,h) in the thalamic and hippocampal region of the post-mortem neonatal human brain. Best3 was highly expressed in hypertrophic astrocytes, where it was observed in the cell bodies and extended into the filamentous processes.

### Expression and Alternative Splicing of Best3 mRNA in the Mouse Brain Tissue Is Changed After HI in the Injured and Non-injured Hemispheres

#### Alternative Splicing of Best3 mRNA

**FIGURE 2:**
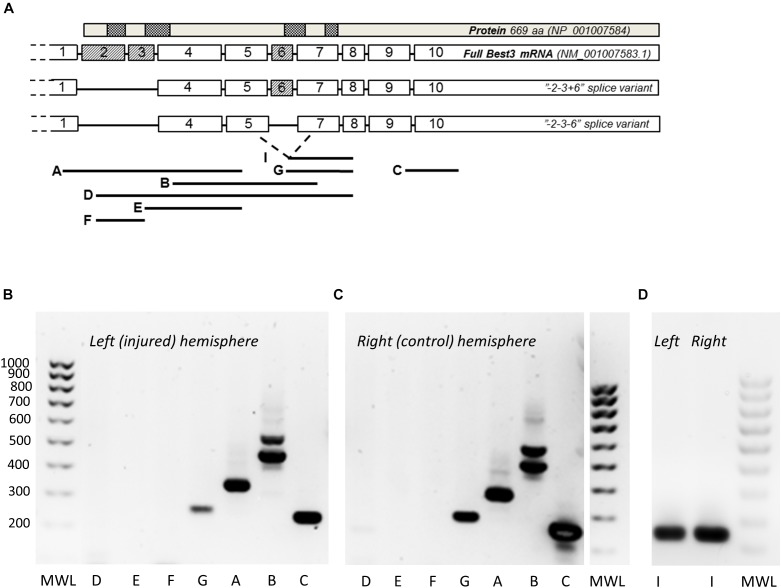
PCR analysis of Best3 mRNA alternative splicing in mouse brain with and without HI injury. Alternative splicing of Best3 mRNA was studied by a set of primers **(A)** spanning the areas of expected splicing of exons 2, 3, and 6 in different combinations as described in detail previously (Golubinskaya et al., 2015). The results of these PCR experiments are presented in **(B–D)**. Total Best3 mRNA can be detected by primer pair C localized in exons 9 and 10 in both healthy **(C)** and injured brain **(B)**. Splice variants of Best3 with exons 2 and 3 present were not detected, neither in healthy brain nor in injury (pairs of primers: A, localized in exons 1 and 5; D in exons 2 and 7; E in exons 3 and 5; F in exons 2 and 3). Exon 6 can be either present or spliced out in Best3 mRNA. Primer pair B spanning exons 4–7 gives two bands, the heavier band corresponds to “+6” and the lighter one to “–6” splice variants. The pair of primers G localized in exons 6 and 8 shows a band corresponding to “+6” splice variant. Primer pair I detects the “–6” variant **(D)**. Primer pairs G and I can potentially detect more than one transcript corresponding to “+6” and “–6” variants depending on possible splicing of exon 10. Positive controls for these primers have been published previously by our group (Golubinskaya et al., 2015). MWL, molecular weight ladder.

The PCR analysis of Best3 mRNA was made by using specifically made primer pairs published previously (Golubinskaya et al., 2015) spanning the expected areas of splicing of exons 2, 3, and 6 (Figure 2A) based on earlier published data on alternative splicing in Best3 mRNA in mouse tissues (Krämer et al., 2004; Srivastava et al., 2008). We did not investigate the possible splicing of exon 10 that recently has been described by Wu et al. (2016), so in our PCR experiments the “-2-3+6” and “-2-3-6” splice variants of mRNA could represent corresponding groups of splice variants where also exon 10 is alternatively spliced. Analysis of the ipsilateral, injured hemisphere and the contralateral non-injured hemisphere (Figure 2B–D) showed the presence of two splice variants of Best3 in both hemispheres: one longer variant where exon 6 was present (“+6” variant) and another shorter one, “-6” variant, where exon 6 was spliced out. This conclusion was made from the observation that primer pair B, covering exons 4–7, gave two bands of corresponding weights. The presence of the “+6” splice isoform was confirmed by the band corresponding to the primer pair G with forward primer placed in exon 6, and the presence of the “-6” isoform by the band corresponding to primer pair I targeting the junction of exons 5 and 7. The “+6” variant was dominating in brain tissue.

At the same time no full-length mRNA for Best3 could be detected in either hemisphere, as exons 2 and 3 were absent in both “-6” and “+6” splice variants. Primer pair A, spanning exons 1 and 5, gave only one band with a weight corresponding to the splice variant with both exons 2 and 3 absent. Neither primer pairs D and E, where one of the primers in each pair was complementary to the sequence in exon 2 or 3, nor primer pair F spanning exons 2 and 3, revealed any band by PCR analysis.

#### Time Course of Changes in Best3 mRNA Expression in the Non-injured Brain Hemisphere After HI

**FIGURE 3:**
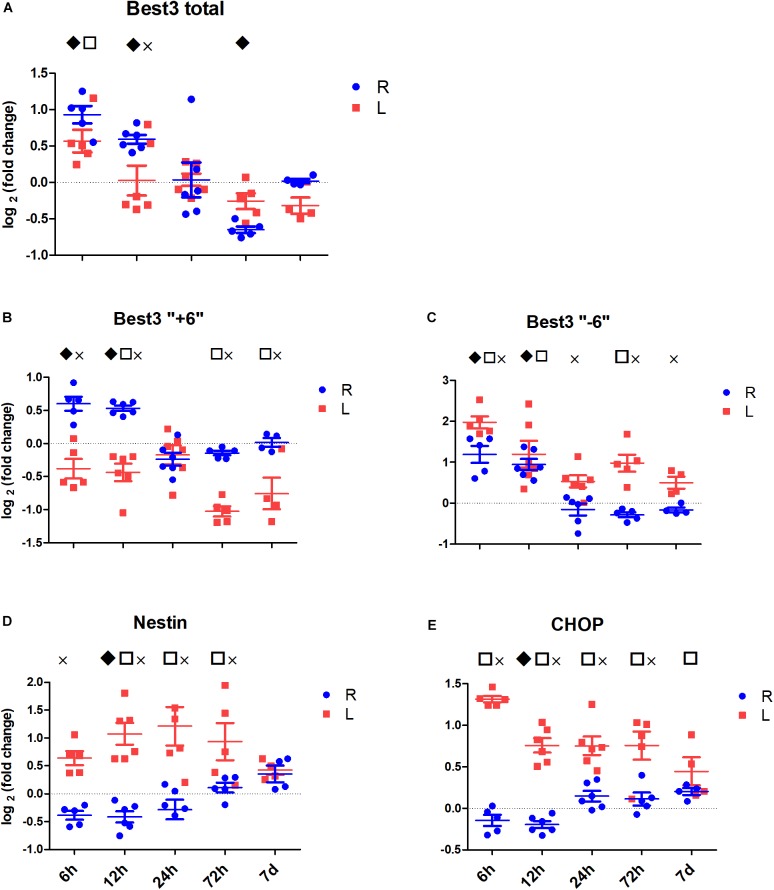
The expression of Best3 mRNA in mouse brain tissue at different time points after HI. Figures show relative expression of the studied mRNAs in uninjured (R, blue) and injured (L, red) hemispheres in relation to the average expression in corresponding hemispheres of sham animals at the respective time points (sham group mean set to zero in the figure). Statistical analysis was performed by mixed-model three-way ANOVA on *C*p values; main effects were based on subsequently estimated marginal means. Symbols above graphs indicate significant difference between uninjured and sham (R vs. sham group at zero, 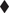), between injured and sham (L vs. sham group at zero, 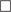), and between uninjured and injured (R vs. L, ×), all symbols indicate P < 0.05. The total mRNA for Best3 increased in both hemispheres at early time points after HI, but returned to control values already 24 h after HI, and even showed a transient decrease at 72 h **(A)**. The long “+6” splice variant increased only in the uninjured hemisphere, while in the injured hemisphere it decreased and stayed decreased through all time point except 24 h **(B)**. The short “–6” splice variant transiently increased at 6 and 12 h, and in the injured hemisphere also again at 72 h **(C)**. Expression of mRNA for nestin **(D)** decreased at early time points in non-injured hemisphere, but returned to control values at 72 h after HI, while it increased in the injured hemisphere during this period to return to control values at 7 days after injury. The ER-stress marker CHOP slightly decreased during the early period after HI in the non-injured hemisphere, but was elevated throughout the studied period in the injured hemisphere **(E)**. A total of 41 pups (26 HI, 15 sham) were used; for details see section “Materials and Methods.” Detailed statistical results are given in the (Supplementary Table S1).

By comparing the right contralateral hemispheres of HI-treated mice to the right hemispheres of sham-operated mice, one can estimate the effect of HI on the uninjured hemisphere (Figure 3, blue marking compared to the zero level). In our experiments HI induced early changes in Best3 mRNA expression in the contralateral (right) hemisphere. Total Best3 (Figure 3A) showed a biphasic response with an early increase at 6 and 12 h and down-regulation at 72 h. The two splice variants followed similar initial time courses, increasing at 6 and 12 h after HI, but after 24 h the values were back to control level and no further changes occurred during the later time points (Figure 3B,C).

Nestin expression was transiently downregulated at 6, 12, and 24 h, returning to its control values at 72 h after HI (Figure 3D). There were no signs of pronounced ER stress in the non-injured brain hemisphere after HI, as expression of the ER-stress marker CHOP did not increase, but rather slightly decreased at the early time points after HI (Figure 3E).

#### Time Course of Changes in Best3 mRNA Expression in Brain Injured After HI

The total effect of HI can be studied by comparing the left ipsilateral (injured) hemisphere of HI-exposed mice with the left hemisphere of sham-operated animals (Figure 3, red marking compared to the zero level). Total mRNA for Best3 increased transiently at 6 h after HI, and then remained at control level (Figure 3A). The long and the short splice variants of Best3 showed opposite changes: the “+6” variant was downregulated (Figure 3B), and “-6” was markedly upregulated (Figure 3C). At 24 h after injury the expression of both splice variants was close to the control values (Figure 3B,C).

Both nestin (Figure 3D) and ER-stress marker CHOP (Figure 3E) were upregulated already at 6 h and stayed upregulated through most of the investigated period. Nestin developed its maximal expression between 12 and 72 h, while CHOP showed maximum expression at 6 h after HI, after which its expression decreased over time toward baseline.

#### Time Course of Injury-Related Changes in Best3 mRNA Expression in the Brain After HI

By comparing the left ipsilateral (injured; Figure 3, red marking) with the right contralateral (non-injured; Figure 3, blue marking) hemisphere in the same animal the changes produced by the development of tissue injury can be estimated. Figure 3 shows that injury reduced the expression of total Best3 mRNA at 12 h only (Figure 3A). Best3 splice variants changed in opposite directions: the long “+6” splice variant was downregulated in a biphasic manner with a transient return to control values at 24 h after HI (Figure 3B), while the expression of the short “-6” splice variant, on the other hand, slightly increased at 6 h and from 24 h onwards compared to the non-injured hemisphere (Figure 3C).

Nestin gradually increased in response to injury, reaching maximal expression between 12 and 24 h, and returning to its control values by 7 days after HI (Figure 3D). Injury was associated with ER stress as indicated by CHOP expression, which was maximally increased at 6 h, and then gradually decreased, returning to its control values at 7 days after HI (Figure 3E). Details of the statistical analysis are given in the Supplementary Table S1.

### ER Stress Alters Expression of Best3 mRNA in Mouse Cultured Astrocytes

**FIGURE 4:**
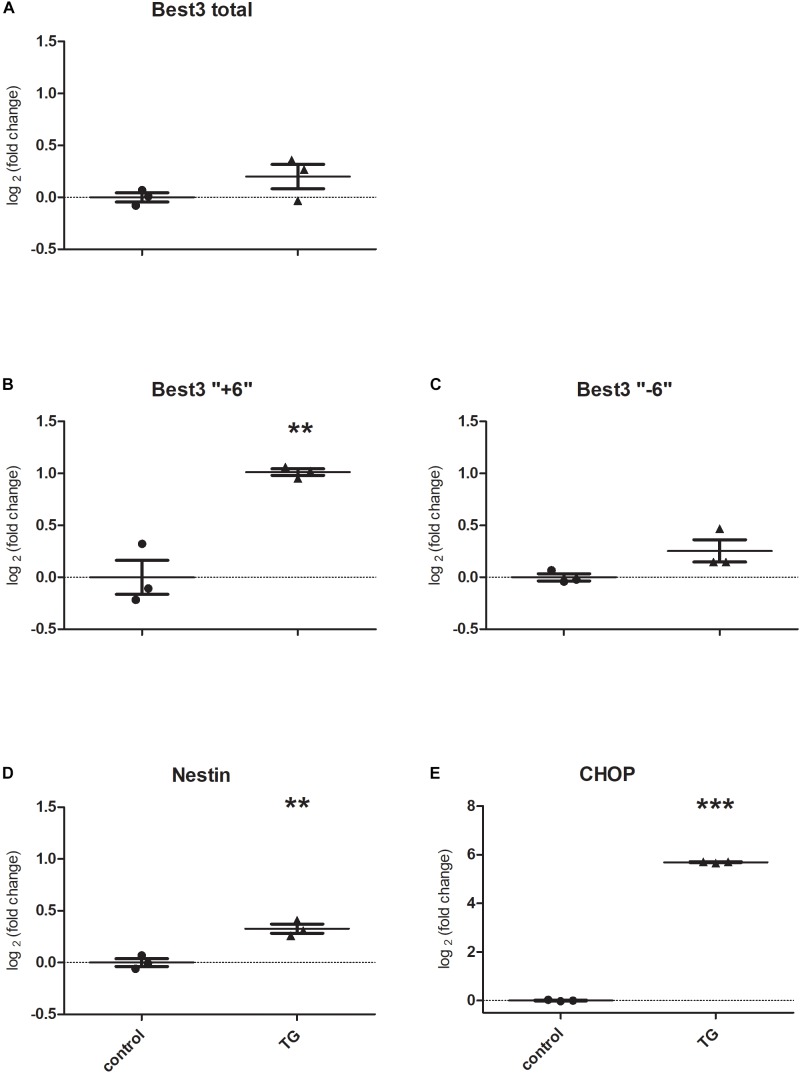
Best3 mRNA is expressed in the primary culture of mouse astrocytes. TG caused very small changes in total Best3 **(A)** and in its short splice variant expression **(C)**, but induced a noticeable increase in expression of the long “+6” splice variant of Best3 **(B)**. TG-treated cells showed a pronounced ER-stress **(E)** and increase in nestin expression **(D)**. ^∗∗^*P* < 0.01, ^∗∗∗^*P* < 0.001 *t*-test control vs. treatment.

The results of the experiments are presented in Figure 4. To confirm that Best3 is altered in astrocytes after injury we investigated mRNA expression for Best3 in cultured astrocytes after inducing ER stress by TG. Before inducing ER stress the “-6” variant was dominating. ER stress caused an increase in expression of total Best3 and both its splice variants (Figure 4A–C). The increase in the long “+6” splice variant was the most pronounced (*P* < 0.01; Figure 4B). Nestin also increased after TG (*P* < 0.01; Figure 4D), and CHOP, as an ER-stress marker, was dramatically upregulated (*P* < 0.001; Figure 4E).

### Best3 Protein Expression Analysis by Western Blot

**FIGURE 5:**
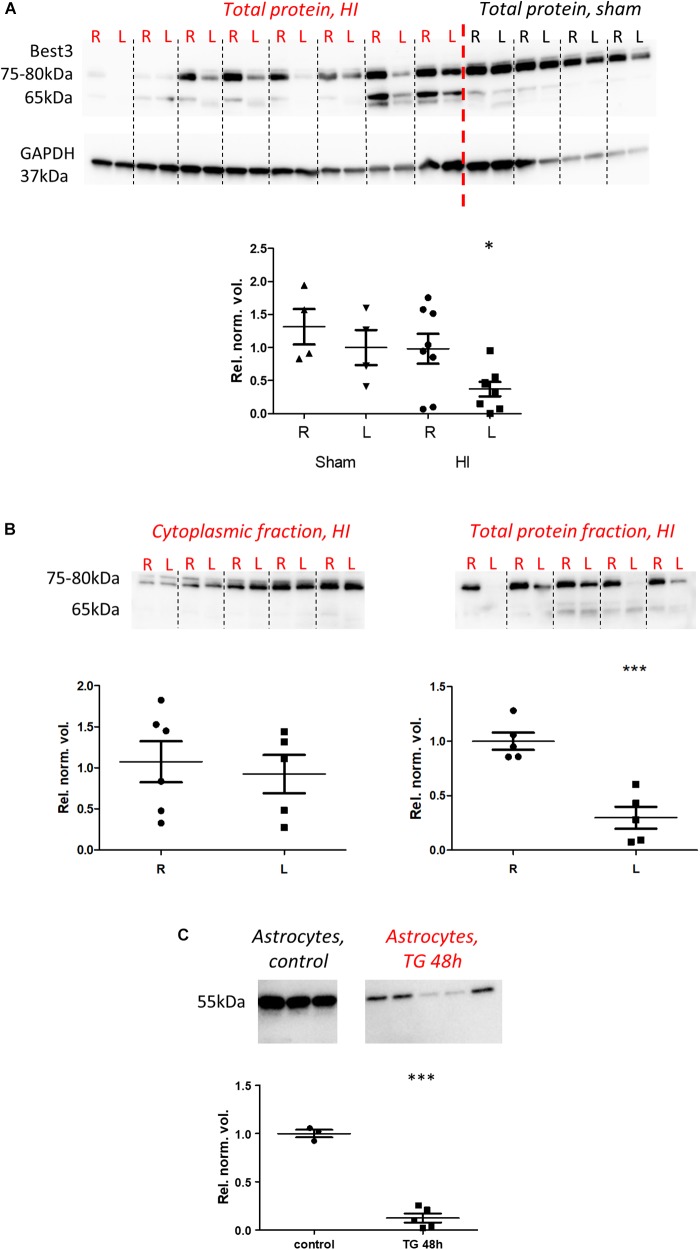
Best3 protein is downregulated in mouse brain tissue and cultured mouse astrocytes after injury. Western blot analysis of brain tissue **(A,B)** and cultured astrocytes **(C)**. Brain specimens from right uninjured (R) and left injured (L) hemispheres were alternately placed on the gels in **(A,B)**; the same was done for sham-operated animals **(A)**. Two neighboring R and L lanes belong to the same animal. In total protein samples from brain **(A)** at approximately 75–80 kDa two bands with close molecular weights could be detected; their density was summarized in the analysis. These bands were reduced in injury. Additional lighter pairs of bands could be observed at approximately 65 kDa. The result of subcellular fractionation of brain tissue is shown in **(B)**, where two bands of approximately 75–80 kDa were detected in the cytoplasmic fraction as well as in the total protein fraction. The content of Best3 in the cytoplasm did not change significantly in injury, while Best3 in the total protein fraction was downregulated. In cultured astrocytes **(C)** treatment with thapsigargin for 48 h (TG 48 h) also caused downregulation of Best3. ^∗^P < 0.05 by two-way ANOVA and estimated marginal means; ^∗∗∗^P < 0.001 *t*-test R vs. L or treated vs. untreated cells. A total of 18 pups (14 HI, 4 sham) were used; for details see section “Materials and Methods.”

Protein analysis by western blot was performed in brain tissue using ARP50108_P050 antibody and in astrocyte culture by Bst-301AP. It revealed Best3-related protein bands in the range of 55–80 kDa (Figure 5). In homogenates of parts of cortex from each hemisphere, typically Best3 staining revealed a pair of bands with molecular weight differing by approximately 2–3 kDa, which might correspond to the predicted difference between “+6” and “-6” splice variants of Best3. As there is no antibody specifically recognizing splice variants of Best3, we calculated the density of both bands together in our analysis.

Lighter pairs of bands at about 65 kDa could often be seen. The appearance of these additional bands likely might be related to the homogenization protocol, by which possibly posttranslational modifications or tightly bound proteins, for example a protein phosphatase (Marmorstein et al., 2002) could be partially removed. It is interesting to note that in cultured astrocytes the Best3-related band had a weight of approximately 52–55 kDa, closer to the theoretically predicted weight for “+6” and “-6” splice variants of Best3. Whether the lower molecular weight in cultured cells relates to a lack of modifications/associations remains to be determined.

Quantification of western blot results showed downregulation of Best3 protein expression in brain cortex and hippocampus after HI (*P* < 0.05) and in cultured mouse astrocytes in ER stress (*P* < 0.001; Figure 5), although this was not apparent in the cytoplasmic fraction of the brain tissue after HI injury (*P* > 0.05). The same result was observed whether Best3-related protein bands were normalized to total protein (Figure 5) or to GAPDH (data not shown).

## Discussion

Best3 protein has previously not been described in the brain, and there are only a few reports where Best3 mRNA was detected in the whole adult mouse brain, although without identification of the cells expressing it (Krämer et al., 2004; Srivastava et al., 2008), and only weakly detected in the normal adult human brain (Stöhr et al., 2002). We show for the first time that Best3 protein and mRNA are expressed in normal and injured brain in newborn mouse pups and in a term infant with white matter gliosis.

Our main focus in this study was to investigate Best3 in cell injury as recent studies suggest a novel role for Best3 in apoptosis and ER-stress. In our mouse experiments we describe for the first time a subpopulation of nestin-positive astrocytes appearing after the HI injury, which expresses Best3 and can be visualized primarily in the penumbra-like area (Figure 1a). These cells have an astrocyte morphology, are positive for GFAP, a classic marker of astrocytes (Figure 1b,d) and for nestin (Figure 1a,c), and do not co-express neuronal or microglial markers. Under normal conditions nestin expression in the brain is more characteristic for progenitor cells than for astrocytes. We cannot exclude the possibility that some of the Best3-positive cells were neural progenitor cells, yet we did not see Best3 expression in the uninjured brain. However, the possibility that progenitor cell proliferation, triggered by injury, contributes to the Best3 expression cannot be ruled out. After injury a subpopulation of activated astrocytes start expressing nestin (Gilyarov, 2008), and these cells have been suggested to be in an early stage of activation preceding hypertrophic changes (Cho et al., 2013). Functionally, these cells have been shown to be proliferating astrocytes that have a positive influence on tissue recovery (Suzuki et al., 2012). The appearance of nestin+/GFAP+ cells has been described in the neonatal rat brain after HI, and these cells are suggested to be in a transition state from nestin-positive radial glia into GFAP-expressing mature astrocytes (Sizonenko et al., 2008). Mouse astrocytes in primary culture in the present study showed a similar pattern of Best3 protein expression as after HI *in vivo*: partial overlap with GFAP and high degree of co-expression with nestin (Figure 1e,f). These results confirm the presence of Best3 in astrocytes, and the pronounced expression of both nestin and Best3 in the majority of cells suggests that astrocytes in culture are to a large extent in an activated state.

We also show that Best3 is expressed in astrocytes in the human term neonatal brain with neuropathology as a result of an acute event leading to intrauterine death (Figure 1g,h). This indicates the relevance of our observations in mice to situations with brain injury in humans. This term neonate very likely had injury to brain cells as a result of different traumatic events leading to death. As a result, the brain tissue shows signs of pronounced gliosis, in particular strong activation of astrocytes, and it was interesting to see expression of Best3 in this clinical case. Best3-positive cells were reminiscent of activated astrocytes by their morphology and developed cell processes. Based on the morphology we can suggest that Best3 can be expressed in a subpopulation of activated astrocytes in situations of neuropathology in neonates. The specific relevance of this discovery to hypoxic-ischemic brain injury in neonates, however, is not determined, and a more detailed study of the co-localization of Best3 with various cell markers is required to characterize the Best3-positive human cells.

In the HI model exposure to hypoxia alone does not induce brain injury, while its combination with ischemia causes appearance of an infarct area and cell apoptosis (Hedtjärn et al., 2002). Total Best3 mRNA was upregulated early after HI in both hemispheres. This change thus is related either to hypoxemia or to some influence of the injury on both hemispheres, whether neural or humoral. Interestingly, the rise in Best3 mRNA lasted longer in the uninjured hemisphere. Whether this relates to that hemisphere coping with the hypoxemia, and the other one succumbing, remains to be investigated.

The alternative splicing of Best3 mRNA after the HI injury in mouse brain was also analyzed. In mouse Best3 mRNA exons 2, 3, and 6 can be spliced out in different combinations in different organs, and the whole brain homogenate from the adult mouse was reported to have two splice variants “-2-3+6” and “-2-3-6”, but not the full-length mRNA (Krämer et al., 2004). Similar results were obtained in our PCR analysis of Best3 splicing in the brains of neonatal mice (Figure 2), and the composition of splice variants was similar in contralateral non-injured and ipsilateral injured brain hemispheres. However, there is also recent data on possible splicing of the C-terminal region of Best3 mRNA and protein in mouse myoblasts (Wu et al., 2016). If this is the case also in brain, the “+6” and “-6” variants seen in our experiments could represent more than one transcript each. A more detailed analysis of alternative splicing of Best3 in situations with brain injury may therefore require RNA sequencing.

In contrast to total Best3, expression of the long “+6” splice variant changed in an injury-related manner, it was downregulated in the injured hemisphere in parallel with developing ER stress, but it was upregulated on the non-injured side (Figure 3). The short “-6” was less injury-dependent and was mostly upregulated in both hemispheres. These changes in splicing started already at 6 h after HI, and it is interesting to note that the changes in expression of both splice variants showed biphasic dynamics: the values always tended to transiently return to control levels 24 h after the injury.

Increased expression of the transcription factor CHOP and nestin mRNA was present in the injured hemisphere only (Figure 3) suggesting pro-apoptotic ER stress in this tissue (Xu et al., 2005), although the degree of apoptosis was not examined in the present study. In non-injured brain tissue CHOP and nestin were actually downregulated after HI, which might be related to compensatory changes in blood flow and is an interesting observation for future investigation.

In mouse astrocyte cell culture (Figure 4) direct induction of strong ER stress by blocking the SERCA pump showed, together with a dramatic increase in CHOP expression, also a tendency for total Best3 mRNA to increase as well as mRNA for both its splice variants, with the most pronounced change in “+6” splice variant expression in contrast to the injury in the brain tissue, where “+6” variant was downregulated. This may indicate that different origins of ER stress can influence Best3 expression differently, but it could also show that cultured astrocytes, being already in an activated state, respond to ER stress differently from astrocytes *in vivo*.

Our results of Best3 mRNA expression show that it is important to study not only the total expression of the gene, but also to analyze individual splice variants, as such proteins can have different functions. Alternative splicing of mRNA can be changed by cell injury and inflammation, and this phenomenon is well described for the protein family Bcl2 (Bcl2, Bcl2L1, Bax, and other), which can act as pro- or antiapoptotic proteins depending on their splicing (Glasgow et al., 2001; Akgul et al., 2004; Miura et al., 2012). A change in the ratio between the antiapoptotic long Bcl-xL and the proapoptotic short Bcl-xS splice variants of one of the members of this family, Bcl2L1 (also called BclX), was also described in neonatal HI injury (Xiao et al., 2012). The results of the present work and of our previous studies (Golubinskaya et al., 2015) reveal a new protein, Best3, alternative splicing of which changes in the situation of tissue injury.

Analysis of Best3 protein expression by western blot showed that the general expression of Best3 protein in brain cortex and hippocampus was reduced after HI injury, and yet in our IHC experiments Best3 appeared in a specific cell population around the injured area. This data suggests that the western blot shows the overall expression in all cell types of the brain and cannot detect an increase in a small sub-population of Best3-positive astrocytes. At the same time expression of Best3 protein was strongly reduced in cultured astrocytes after 48 h with thapsigargin treatment. Both HI injury and thapsigargin treatment caused ER stress, judging from the increase in CHOP. The Best3 protein was downregulated in cultured cells during ER stress, which calls into question whether Best3 is actually involved in resolving the ER-stress response in astrocytes. On the other hand, the severity of ER stress may differ between the brain astrocytes in HI and thapsigargin-treated cultured astrocytes, where thapsigargin presumably is a much stronger inducer of ER stress or induces ER stress by different mechanisms. Another reason may be that ER stress as such may cause downregulation of Best3 protein, whereas signals related to tissue injury and coming from other types of the cells cause upregulation of Best3 in astrocytes close to the injured area. It is also interesting to note that Best3 protein levels did not change significantly in the cytoplasmic fraction of brain cortex, suggesting the reduction in protein must occur in other compartments of the cells.

In summary, our study demonstrates expression of Best3 in a nestin-positive subpopulation of astrocytes in neonatal mouse brain after hypoxic-ischemic injury, with a general suppression of its expression in the cortex as a whole. Best3 also shows localized expression in astrocyte-like cells in human neonatal brain with pathologies. Analysis of alternative splicing for Best3 mRNA in mouse brain after HI injury showed that even if total expression of Best3 mRNA may not show much change, its splice variants respond differently and thus might have different roles in development of injury in brain tissue. The shorter splice variants are unlikely to function as ion channels (Srivastava et al., 2008) although they may have both intracellular and membrane localization. Their function is so far unknown. If Best3 actually functions as a regulator of apoptosis, it is possible that this property is associated with one variant as it is with Bcl-X, however, this is highly speculative and needs further investigation. Best3 expression in astrocytes near injury in the neonatal brain is a novel and important finding. As a marker for a certain cell population it can help understanding mechanisms of brain injury, where glial cells play an important role. A definitive determination of the role for Best3 in pathophysiology hinges upon the development of a knock-out model.

## Author Contributions

VG, HG, CM, and HN designed the study. VG, RV, and JW-A collected the data. VG, RV, JW-A, CM, and HN analyzed the data. VG, CM, and HN drafted the manuscript. All authors revised and approved the manuscript.

## Conflict of Interest Statement

The authors declare that the research was conducted in the absence of any commercial or financial relationships that could be construed as a potential conflict of interest.

## References

[B1] Akgul C. Moulding D. A. Edwards S. W. (2004). Alternative splicing of Bcl-2-related genes: functional consequences and potential therapeutic applications. *Cell. Mol. Life Sci.* 61 2189–2199. 10.1007/s00018-004-4001-7 15338051 PMC11138917

[B2] Blomgren K. Zhu C. Wang X. Karlsson J.-O. Leverin A.-L. Bahr B. A. (2001). Synergistic activation of caspase-3 by m-calpain after neonatal hypoxia-ischemia a mechanism of “pathological apoptosis”? *J. Biol. Chem.* 276 10191–10198. 10.1074/jbc.M007807200 11124942

[B3] Chavez-Valdez R. Flock D. L. Martin L. J. Northington F. J. (2016). Endoplasmic reticulum pathology and stress response in neurons precede programmed necrosis after neonatal hypoxia-ischemia. *Int. J. Dev. Neurosci.* 48 58–70. 10.1016/j.ijdevneu.2015.11.007 26643212 PMC4718855

[B4] Cho J. M. Shin Y. J. Park J. M. Kim J. Lee M. Y. (2013). Characterization of nestin expression in astrocytes in the rat hippocampal CA1 region following transient forebrain ischemia. *Anat. Cell Biol.* 46 131–140. 10.5115/acb.2013.46.2.131 23869260 PMC3713277

[B5] Cox B. Emili A. (2006). Tissue subcellular fractionation and protein extraction for use in mass-spectrometry-based proteomics. *Nat. Protoc.* 1 1872–1878. 10.1038/nprot.2006.273 17487171

[B6] de Vellis J. Ghiani C. A. Wanner I. B. Cole R. (2010). “Preparation of normal and reactive astrocyte cultures,” in *Protocols for Neural Cell Culture Springer Protocols Handbooks*, ed. Doering L. C. (New York, NY: Humana Press), 193–215.

[B7] DeGracia D. J. Montie H. L. (2004). Cerebral ischemia and the unfolded protein response. *J. Neurochem.* 91 1–8. 10.1111/j.1471-4159.2004.02703.x 15379881

[B8] Duran C. Thompson C. H. Xiao Q. Hartzell H. C. (2010). Chloride channels: often enigmatic, rarely predictable. *Annu. Rev. Physiol.* 72 95–121. 10.1146/annurev-physiol-021909-135811 19827947 PMC2851227

[B9] Ek C. J. D’Angelo B. Baburamani A. A. Lehner C. Leverin A.-L. Smith P. L. (2015). Brain barrier properties and cerebral blood flow in neonatal mice exposed to cerebral hypoxia-ischemia. *J. Cereb. Blood Flow Metab.* 35 818–827. 10.1038/jcbfm.2014.255 25627141 PMC4420855

[B10] Gilyarov A. V. (2008). Nestin in central nervous system cells. *Neurosci. Behav. Physiol.* 38 165–169. 10.1007/s11055-008-0025-z 18197384

[B11] Glasgow J. N. Qiu J. Rassin D. Grafe M. Wood T. Perez-Pol J. R. (2001). Transcriptional regulation of the BCL-X gene by NF-kappaB is an element of hypoxic responses in the rat brain. *Neurochem. Res.* 26 647–659. 10.1023/A:1010987220034 11519724

[B12] Golubinskaya V. Elvin J. Ebefors K. Gustafsson H. Mallard C. Nyström J. (2015). Bestrophin-3 is differently expressed in normal and injured mouse glomerular podocytes. *Acta Physiol.* 214 481–496. 10.1111/apha.12516 25912364

[B13] Han K. S. Woo J. Park H. Yoon B. J. Choi S. Lee C. J. (2013). Channel-mediated astrocytic glutamate release via Bestrophin-1 targets synaptic NMDARs. *Mol. Brain* 6:4. 10.1186/1756-6606-6-4 23324492 PMC3577500

[B14] Hedtjärn M. Leverin A.-L. Eriksson K. Blomgren K. Mallard C. Hagberg H. (2002). Interleukin-18 involvement in hypoxic–ischemic brain injury. *J. Neurosci.* 22 5910–5919. 10.1523/JNEUROSCI.22-14-05910.2002 12122053 PMC6757918

[B15] Jiang L. Liu Y. Ma M. M. Tang Y. B. Zhou J. G. Guan Y. Y. (2013). Mitochondria dependent pathway is involved in the protective effect of bestrophin-3 on hydrogen peroxide-induced apoptosis in basilar artery smooth muscle cells. *Apoptosis* 18 556–565. 10.1007/s10495-013-0828-4 23468120

[B16] Johnson G. G. White M. C. Wu J.-H. Vallejo M. Grimaldi M. (2014). The deadly connection between endoplasmic reticulum, Ca^2+^, protein synthesis, and the endoplasmic reticulum stress response in malignant glioma cells. *Neuro Oncol.* 16 1086–1099. 10.1093/neuonc/nou012 24569545 PMC4096176

[B17] Krämer F. Stöhr H. Weber B. H. (2004). Cloning and characterization of the murine Vmd2 RFP-TM gene family. *Cytogenet. Res.* 105 107–114. 10.1159/000078016 15218265

[B18] Lee S. Yoon B. E. Berglund K. Oh S. J. Park H. Shin H. S. (2010). Channel-mediated tonic GABA release from glia. *Science* 330 790–796. 10.1126/science.1184334 20929730

[B19] Lee W. K. Chakraborty P. K. Roussa E. Wolff N. A. Thevenod F. (2012). ERK1/2-dependent bestrophin-3 expression prevents ER-stress-induced cell death in renal epithelial cells by reducing CHOP. *Biochim. Biophys. Acta* 1823 1864–1876. 10.1016/j.bbamcr.2012.06.003 22705154

[B20] Lehotský J. Urban P. Pavlíková M. Tatarková Z. Kaminska B. Kaplán P. (2009). Molecular mechanisms leading to neuroprotection/ischemic tolerance: effect of preconditioning on the stress reaction of endoplasmic reticulum. *Cell. Mol. Neurobiol.* 29 917–925. 10.1007/s10571-009-9376-4 19283468 PMC11506296

[B21] Leonard A. Paton A. W. El-Quadi M. Paton J. C. Fazal F. (2014). Preconditioning with endoplasmic reticulum stress ameliorates endothelial cell inflammation. *PLoS One* 9:e110949. 10.1371/journal.pone.0110949 25356743 PMC4214695

[B22] Lund S. Christensen K. V. Hedtjärn M. Mortensen A. L. Hagberg H. Falsig J. (2006). The dynamics of the LPS triggered inflammatory response of murine microglia under different culture and in vivo conditions. *J. Neuroimmunol.* 180 71–87. 10.1016/j.jneuroim.2006.07.007 16996144

[B23] Marmorstein L. Y. McLaughlin P. J. Stanton J. B. Yan L. Crabb J. W. Marmorstein A. D. (2002). Bestrophin interacts physically and functionally with protein phosphatase 2A. *J. Biol. Chem.* 277 30591–30597. 10.1074/jbc.M204269200 12058047

[B24] Matchkov V. V. Larsen P. Bouzinova E. V. Rojek A. Boedtkjer D. M. Golubinskaya V. (2008). Bestrophin-3 (vitelliform macular dystrophy 2-like 3 protein) is essential for the cGMP-dependent calcium-activated chloride conductance in vascular smooth muscle cells. *Circ. Res.* 103 864–872. 10.1161/CIRCRESAHA.108.178517 18776041

[B25] Miura K. Fujibuchi W. Unno M. (2012). Splice variants in apoptotic pathway. *Exp. Oncol.* 34 212–217. 23070006

[B26] Oh S. J. Han K. S. Park H. Woo D. H. Kim H. Y. Traynelis S. F. (2012). Protease activated receptor 1-induced glutamate release in cultured astrocytes is mediated by Bestrophin-1 channel but not by vesicular exocytosis. *Mol. Brain* 5:38. 10.1186/1756-6606-5-38 23062602 PMC3539998

[B27] Oh S.-J. Lee C. J. (2017). Distribution and function of the bestrophin-1 (Best1) channel in the brain. *Exp. Neurobiol.* 26 113–121. 10.5607/en.2017.26.3.113 28680296 PMC5491579

[B28] Park H. Oh S. J. Han K. S. Woo D. H. Park H. Mannaioni G. (2009). Bestrophin-1 encodes for the Ca^2+^-activated anion channel in hippocampal astrocytes. *J. Neurosci.* 29 13063–13073. 10.1523/JNEUROSCI.3193-09.2009 19828819 PMC2825675

[B29] Rice J. E. III Vannucci R. C. Brierley J. B. (1981). The influence of immaturity on hypoxic-ischemic brain damage in the rat. *Ann. Neurol.* 9 131–141. 10.1002/ana.410090206 7235629

[B30] Sheldon R. A. Sedik C. Ferriero D. M. (1998). Strain-related brain injury in neonatal mice subjected to hypoxia-ischemia. *Brain Res.* 810 114–122. 10.1016/S0006-8993(98)00892-0 9813271

[B31] Singh K. Han K. Tilve S. Wu K. Geller H. M. Sack M. N. (2018). Parkin targets NOD2 to regulate astrocyte endoplasmic reticulum stress and inflammation. *Glia* 66 2427–2437. 10.1002/glia.23482 30378174 PMC6275110

[B32] Sizonenko S. V. Camm E. J. Dayer A. Kiss J. Z. (2008). Glial responses to neonatal hypoxic-ischemic injury in the rat cerebral cortex. *Int. J. Dev. Neurosci.* 26 37–45. 10.1016/j.ijdevneu.2007.08.014 17942266

[B33] Song W. Yang Z. He B. (2014). Bestrophin 3 ameliorates TNFα-induced inflammation by inhibiting NF-κB activation in endothelial cells. *PLoS One* 9:e111093. 10.1371/journal.pone.0111093 25329324 PMC4203846

[B34] Srivastava A. Romanenko V. G. Gonzalez-Begne M. Catalan M. A. Melvin J. E. (2008). A variant of the Ca^2+^-activated Cl channel Best3 is expressed in mouse exocrine glands. *J. Membr. Biol.* 222 43–54. 10.1007/s00232-008-9098-4 18414923

[B35] Stöhr H. Marquardt A. Nanda I. Schmid M. Weber B. H. (2002). Three novel human VMD2-like genes are members of the evolutionary highly conserved RFP-TM family. *Eur. J. Hum. Genet.* 10 281–284. 10.1038/sj.ejhg.5200796 12032738

[B36] Supramaniam V. Vontell R. Srinivasan L. Wyatt-Ashmead J. Hagberg H. Rutherford M. (2013). Microglia activation in the extremely preterm human brain. *Pediatr. Res.* 73 301–309. 10.1038/pr.2012.186 23364172

[B37] Suzuki T. Sakata H. Kato C. Connor J. A. Morita M. (2012). Astrocyte activation and wound healing in intact-skull mouse after focal brain injury. *Eur. J. Neurosci.* 36 3653–3664. 10.1111/j.1460-9568.2012.08280.x 23013365 PMC5394426

[B38] Vannucci S. J. Hagberg H. (2004). Hypoxia-ischemia in the immature brain. *J. Exp. Biol.* 207 3149–3154. 10.1242/jeb.01064 15299036

[B39] Vontell R. Supramaniam V. Thornton C. Wyatt-Ashmead J. Mallard C. Gressens P. (2013). Toll-like receptor 3 expression in glia and neurons alters in response to white matter injury in preterm infants. *Dev. Neurosci.* 35 130–139. 10.1159/000346158 23548575 PMC3826123

[B40] Vontell R. Supramaniam V. Wyatt-Ashmead J. Gressens P. Rutherford M. Hagberg H. (2015). Cellular mechanisms of toll-like receptor-3 activation in the thalamus are associated with white matter injury in the developing brain. *J. Neuropathol. Exp. Neurol.* 74 273–285. 10.1097/NEN.0000000000000172 25668563 PMC4327391

[B41] Wu L. Sun Y. Ma L. Zhu J. Zhang B. Pan Q. (2016). A C-terminally truncated mouse Best3 splice variant targets and alters the ion balance in lysosome-endosome hybrids and the endoplasmic reticulum. *Sci. Rep.* 6:27332. 10.1038/srep27332 27265833 PMC4893618

[B42] Xiao Q. Ford A. L. Xu J. Yan P. Lee K. Y. Gonzales E. (2012). Bcl-x pre-mRNA splicing regulates brain injury after neonatal hypoxia-ischemia. *J. Neurosci.* 32 13587–13596. 10.1523/JNEUROSCI.2617-12.2012 23015448 PMC3482490

[B43] Xu C. Bailly-Maitre B. Reed J. C. (2005). Endoplasmic reticulum stress: cell life and death decisions. *J. Clin. Invest.* 115 2656–2664. 10.1172/JCI26373 16200199 PMC1236697

[B44] Zhu C. Wang X. Xu F. Bahr B. A. Shibata M. Uchiyama Y. (2005). The influence of age on apoptotic and other mechanisms of cell death after cerebral hypoxia–ischemia. *Cell Death Differ.* 12 162–176. 10.1038/sj.cdd.4401545 15592434

