# Supplementary material for: Bestrophin-3 Expression in a Subpopulation of Astrocytes in the Neonatal Brain After Hypoxic-Ischemic Injury
Source: Front Physiol. 2019 Jan 29;10:23. doi: 10.3389/fphys.2019.00023 (PMC6362097; doi:10.3389/fphys.2019.00023)
Supplement: Supplementary file 1 [file Image_1.pdf]

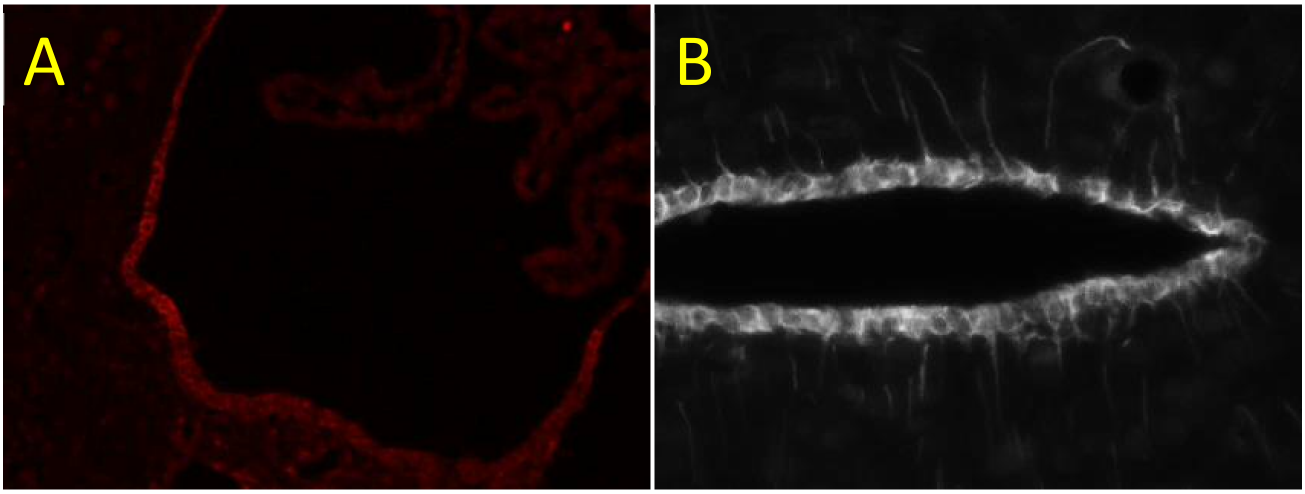

Fig. S1. Expression of Best3 in the healthy brain of newborn mice.
Immunofluorescent detection of Best3 in the brain of 12-days old mouse showed the expression of Best3 in ependymal cells in the wall of the third brain ventricle (A – dorsal part, B – ventral part of the third ventricle). Best3 staining is red (A, magnification 20x) or white (B, magnification 40x). Under normal conditions Best3 protein was seen in ependymal cells in the wall of the brain ventricles, and PCR analysis showed presence of Best3 mRNA in normal brain homogenate (Fig.2). In our initial experiments we also saw expression of Best3 protein in ependymal cells in healthy brains of adult mice and rats. However, the immunohistochemical experiments did not reveal differences in Best3 expression in ependymocytes in injured compared to control hemispheres. Thus the expression of Best3 in ependymocytes does not appear to be injury related.
