# Supplementary material for: Bestrophin-3 Expression in a Subpopulation of Astrocytes in the Neonatal Brain After Hypoxic-Ischemic Injury
Source: Front Physiol. 2019 Jan 29;10:23. doi: 10.3389/fphys.2019.00023 (PMC6362097; doi:10.3389/fphys.2019.00023)
Supplement: Supplementary file 2 [file Image_2.pdf]

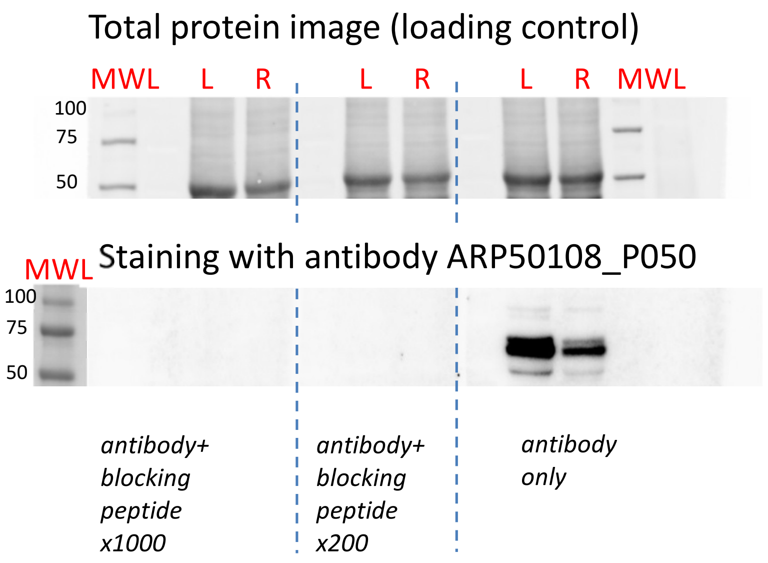


**Fig. S2. Western blot control of specificity of antibody binding by blocking peptide competition assay.**

The appropriate blocking peptide (AAP50108 (Aviva Systems Biology, USA) was used following the manufacturer's protocol to determine antibody specificity. Similar quantities of sample (total protein from brain cortex left (L) and right (R) hemispheres) were transferred to the Western blot membrane (top panel); the membrane was cut into three pieces that were exposed to Best3 antibody either alone or pre-absorbed to either 200- or 1000-fold molar excess of blocking peptide (bottom panel). MWL = molecular weight ladder.
