# Supplementary material for: Bestrophin-3 Expression in a Subpopulation of Astrocytes in the Neonatal Brain After Hypoxic-Ischemic Injury
Source: Front Physiol. 2019 Jan 29;10:23. doi: 10.3389/fphys.2019.00023 (PMC6362097; doi:10.3389/fphys.2019.00023)
Supplement: Supplementary file 3 [file Table_1.pdf]

Table S1

The table shows statistics for the data in Fig. 3. Data were analyzed by mixed-model three-way ANOVA on Cp values. The table shows significance data for main effects and interactions as well as for estimated marginal means. In the table, side means comparison between uninjured (right) and injured (left) hemispheres, treatment is comparison between hypoxia-ischemia and sham, and period is comparison between the various time points. R vs sham shows comparison of estimated marginal means between uninjured (right) hemisphere for hypoxia-ischemia and sham for the various time points, L vs sham the same for the left (injured) ventricle, and R vs L a similar comparison between the two hemispheres in hypoxia-ischemia. df = degrees of freedom, F = Fisher's F value, P = significance.

Fig 3a Best3 total

		**df**	**F**	**P**
**side**		1,31	1.071	0.309
**treatment**		1,31	1.200	0.282
**period**		4,31	5.300	0.002
**side x treatment**		1,31	1.071	0.309
**side x period**		4,31	1.086	0.380
**side x treatment x period**		4,31	1.086	0.380
**R vs sham**	6h	1,31	10.138	0.003
	12h	1,31	5.237	0.029
	24h	1,31	0.015	0.902
	72h	1,31	4.938	0.034
	7d	1,31	0.003	0.954
**L vs sham**	6h	1,31	5.177	0.030
	12h	1,31	0.014	0.908
	24h	1,31	0.023	0.881
	72h	1,31	1.060	0.311
	7d	1,31	1.166	0.289
**R vs L**	6h	1,31	2.518	0.123
	12h	1,31	7.357	0.011
	24h	1,31	0.000	0.994
	72h	1,31	2.952	0.096
	7d	1,31	1.771	0.193

Fig 3b Best3 "+6"

		df	F	P
**side**		1,31	32.111	0.000
**treatment**		1,31	12.158	0.001
**period**		4,31	5.328	0.002
**side x treatment**		1,31	32.111	0.000
**side x period**		4,31	2.747	0.046
**side x treatment x period**		4,31	2.747	0.046
**R vs sham**	6h	1,31	19.054	0.000
	12h	1,31	19.016	0.000
	24h	1,31	3.165	0.085
	72h	1,31	1.083	0.306
	7d	1,31	0.011	0.916
**L vs sham**	6h	1,31	2.841	0.102
	12h	1,31	4.803	0.036
	24h	1,31	0.616	0.438
	72h	1,31	20.610	0.000
	7d	1,31	7.947	0.008
**R vs L**	6h	1,31	33.903	0.000
	12h	1,31	39.548	0.000
	24h	1,31	0.187	0.669
	72h	1,31	27.160	0.000
	7d	1,31	16.693	0.000

Fig 3c Best "-6"

		df	F	P
**side**		1,31	34.843	0.000
**treatment**		1,31	35.851	0.000
**period**		4,31	6.359	0.001
**side x treatment**		1,31	34.842	0.000
**side x period**		4.31	1.945	0.128
**side x treatment x period**		4,31	1.945	0.128
**R vs sham**	6h	1,31	29.778	0.000
	12h	1,31	23.979	0.000
	24h	1,31	0.539	0.468
	72h	1,31	1.698	0.202
	7d	1,31	0.408	0.528
**L vs sham**	6h	1,31	34.744	0.000
	12h	1,31	16.287	0.000
	24h	1,31	2.687	0.111
	72h	1,31	8.511	0.007
	7d	1,31	1.584	0.218
**R vs L**	6h	1,31	22.044	0.000
	12h	1,31	2.717	0.109
	24h	1,31	20.224	0.000
	72h	1,31	56.865	0.000
	7d	1,31	12.645	0.001

Fig.3d Nestin

		df	F	P
**side**		1,31	30.520	0.000
**treatment**		1,31	13.788	0.001
**period**		4,31	0.497	0.738
**side x treatment**		1,31	30.520	0.000
**side x period**		4,31	1.935	0.129
**side x treatment x period**		4,31	1.935	0.129
**R vs sham**	6h	1,31	3.939	0.056
	12h	1,31	5.788	0.022
	24h	1,31	2.248	0.144
	72h	1,31	0.325	0.572
	7d	1,31	2.353	0.135
**L vs sham**	6h	1,31	3.057	0.090
	12h	1,31	11.024	0.002
	24h	1,31	11.689	0.002
	72h	1,31	6.530	0.016
	7d	1,31	0.959	0.335
**R vs L**	6h	1,31	18.294	0.000
	12h	1,31	42.137	0.000
	24h	1,31	46.489	0.000
	72h	1,31	11.769	0.002
	7d	1,31	0.068	0.796

Fig 3e CHOP

		df	F	P
**side**		1,31	71.304	0.000
**treatment**		1,31	89.429	0.000
**period**		4,31	1.576	0.205
**side x treatment**		1,31	71.305	0.000
**side x period**		4,31	4.486	0.006
**side x treatment x period**		4,31	4.486	0.006
**R vs sham**	6h	1,31	1.891	0.179
	12h	1,31	4.396	0.044
	24h	1,31	2.055	0.162
	72h	1,31	1.137	0.295
	7d	1,31	2.611	0.116
**L vs sham**	6h	1,31	60.668	0.000
	12h	1,31	25.864	0.000
	24h	1,31	21.270	0.000
	72h	1,31	20.047	0.000
	7d	1,31	4.948	0.034
**R vs L**	6h	1,31	136.073	0.000
	12h	1,31	69.806	0.000
	24h	1,31	28.219	0.000
	72h	1,31	26.390	0.000
	7d	1,31	3.006	0.093
